# Riboflavin-LSD1 axis participates in the in vivo tumor-associated macrophage morphology in human colorectal liver metastases

**DOI:** 10.1007/s00262-024-03645-1

**Published:** 2024-03-02

**Authors:** Cristiana Soldani, Giulia De Simone, Michela Anna Polidoro, Aurelia Morabito, Barbara Franceschini, Federico Simone Colombo, Achille Anselmo, Flavio Milana, Ana Lleo, Guido Torzilli, Roberta Pastorelli, Matteo Donadon, Laura Brunelli

**Affiliations:** 1 Laboratory of Hepatobiliary Immunopathology, IRCCS Humanitas Research Hospital, Via Alessandro Manzoni 56, 20089 Rozzano, Milan, Italy; 2 Laboratory of Metabolites and Proteins in Translational Research, Istituto di Ricerche Farmacologiche Mario Negri IRCCS, Via Mario Negri 2, 20156 Milan, Italy; 3 Department of Biotechnologies and Biosciences, Università degli Studi Milano Bicocca, Milan, Italy; 4 Department of Electronics, Information and Bioengineering, Politecnico di Milano, Milan, Italy; 5 Foundation IRCCS Ca’ Granda Ospedale Maggiore Policlinico, Struttura Complessa di Patologia Clinica, Laboratorio di Citometria, Milano, Italy; 6 Flow Cytometry Resource, Advanced Cytometry Technical Application Laboratory, IRCCS Ospedale San Raffaele, Milano, Italy; 7 Department of Biomedical Sciences, Humanitas University, Via Rita Levi Montalcini 4, Pieve Emanuele - Milan, Italy; 8 Division of Internal Medicine and Hepatology, Department of Gastroenterology, IRCCS Humanitas Research Hospital, Rozzano, Milan, Italy; 9 Division of Hepatobiliary and General Surgery, Department of Surgery, IRCCS Humanitas Research Hospital, Via Manzoni 56, 20089 Rozzano, Milan, Italy; 10 Department of Health Sciences, Università del Piemonte Orientale, Novara, Italy; 11 Department of General Surgery, University Maggiore Hospital, Novara, Italy

**Keywords:** Colorectal liver metastasis, Tumor-associated macrophages, Histone lysine-specific demethylase 1, Riboflavin, Tumor microenvironment

## Abstract

Tumor-associated macrophages (TAMs) are key components of the tumor microenvironment (TME). In colorectal liver metastasis (CLM), TAM morphology correlates with prognosis, with smaller TAMs (S-TAMs) conferring a more favorable prognosis than larger TAMs (L-TAMs). However, the metabolic profile of in vivo human TAM populations remains unknown. Multiparametric flow cytometry was used to freshly isolate S- and L-TAMs from surgically resected CLM patients (*n* = 14S-, 14L-TAMs). Mass spectrometry-based metabolomics analyses were implemented for the metabolic characterization of TAM populations. Gene expression analysis and protein activity were used to support the biochemical effects of the enzyme–substrate link between riboflavin and (lysine-specific demethylase 1A, LSD1) with TAM morphologies. L-TAMs were characterized by a positive correlation and a strong association between riboflavin and TAM morphologies. Riboflavin in both L-TAMs and in-vitro M2 polarized macrophages modulates LSD1 protein expression and activity. The inflammatory stimuli promoted by TNFα induced the increased expression of riboflavin transporter SLC52A3 and LSD1 in M2 macrophages. The modulation of the riboflavin-LSD1 axis represents a potential target for reprogramming TAM subtypes, paving the way for promising anti-tumor therapeutic strategies.

**Supplementary Information:**

The online version contains supplementary material available at 10.1007/s00262-024-03645-1.

## Introduction

Tumor-associated macrophages (TAMs) are one of the most common immune cells that populate the tumor microenvironment (TME). Based on the tumor type, stages, and grades, TAMs can acquire different functions and properties, implicated in promoting tumor growth, progression, and metastases [1]. Their clinical relevance has been reported in large cohorts of cancer patients, highlighting TAMs as prognostic indicators of cancer progression and patient prognosis [2]. Generally, high TAM content was found to be associated with anti-inflammatory properties correlated with poor prognosis, as well as with a broad spectrum of metabolic features [3]. Intriguingly, in colorectal liver metastasis (CLM), the morphometric characterization of TAMs can serve as a simple readout of their diversity and allows to reliably stratify patient outcomes and predict disease recurrence after hepatectomy for CLMs [4].

Modifications in macrophage shapes lead to changes in their polarization states, emphasizing the correlation between TAM function and morphology [5]. Notably, M1-like TAMs present a flattened appearance, while M2-like TAMs exhibit a larger, elongated form [6–10]. The activated phenotype (M1 or M2) in solid tumors, however, remains poorly understood. Typically, the M1-like TAMs are linked to early tumor stages and anti-tumor activity, while the pro-inflammatory and immunosuppressive actions of M2-like TAMs predict a worse prognosis across various solid tumor types [9–11].

To date, limited knowledge is available on the metabolic asset of the in vivo human M1- and M2-like TAMs, mainly due to the use of in vitro polarized TAMs [6, 7, 12]. These studies identified different metabolic features among TAMs, highlighting an augmented glycolysis and oxidative phosphorylation (OXPHOS) activity in M2-like TAMs compared to the M1-like ones. Conversely, other authors described that M2 macrophages were characterized by a poor glycolytic profile with higher arginine metabolism and preferential fatty acid oxidation (oxidative glucose metabolism), glutamine metabolism, and OXPHOS [8, 13–15]. The wide spectrum of metabolic features of TAMs may be ascribed to their ability to adapt the metabolism based on different microenvironments (e.g., nutrient, oxygen, and lipid availability), in which they are exposed [7]. Macrophage polarization is defined as the acquisition of an activation state, which could be altered upon multiple signals within the TME, due to the macrophage plasticity [16]. Indeed, pathogens or inflammatory signals, derived from the surrounding cells, can influence macrophage differentiation toward the acquisition of new functions by rapidly modulating the expression of several key genes [17]. In this contest, the mass-spectrometry-based metabolomics approach, capable of acquiring thousands of small molecules from biological matrices, has been demonstrated to identify metabolic dysregulation.

In this study, we profiled by mass spectrometry the metabolic characteristics of patient-derived freshly sorted TAM populations coexisting within CLM, to gain novel insights into the relationship between the in vivo TAMs metabolic asset, their morphologies, and their functions. The identification of the key metabolic determinants, able to discriminate Small (S-) TAMs and Large (L-) TAMs will pave the way for better patient stratification. Furthermore, a deeper understanding of the mechanisms underlying TAM morphology and their polarization state could allow to manipulate their phenotype by targeting the cancer inflammatory microenvironment and developing new therapeutic strategies.

## Material and methods

### Patients

The study included fourteen patients who underwent surgery at the IRCCS Humanitas Research Hospital between 2020 and 2022 for CLM (Table 1). The study protocol, submitted to the international clinical trial registry (ClinicalTrials.gov registration number NCT03888638), was designed to identify macrophage-related morphological features associated with distinct clinical outcomes. Written informed consent was obtained from each patient included in the study. The study protocol was in accordance with the ethical guidelines established in the 1975 Declaration of Helsinki and compliant with the procedures of the local ethical committee of the IRCCS Humanitas Research Hospital (registration number 282/19).

**Table 1:** Demographic and clinical characteristics of the CLM patients used in this study

Variables	*N* = 14
Sex, male, *n* (%)	9 (64.3)
Age, year, median (IQR)	65.4 (53.6–74.4)
Size of CLMs, median (IQR)	3.50 (1.87–5.75)
Number of CLMs, median (IQR)	5.5 (2.5–12.0)
Bilobar disease, *n* (%)	9 (64.3)
Preoperative CEA (ng/ml), median (IQR)	18.7 (4.7–155.9)
Preoperative CA19.9 (IU/ml), median (IQR)	27.2 (7.9–307.1)
Grading of the primary tumor, *n* (%)	
G2–G3	10 (71.4)
Staging of the primary tumor, *n* (%)	
T3–4	9 (64.3)
N+	10 (71.4)
Synchronous presentation, *n* (%)	12 (85.7)
Site of the primary tumor, *n* (%)	
Colon	9 (64.3)
Rectum	5 (35.7)
Neoadjuvant chemotherapy, *n* (%)	11 (78.6)
Chemotherapy lines, median (IQR)	1.00 (0.75–1.00)
Type of chemotherapy, *n* (%)	
5-FU based	3 (21.4)
Oxaliplatin based	8 (57.1)
Irinotecan based	3 (21.4)
+ Anti-VEGF	6 (42.9)
+ Anti-EGFR	3 (21.4)
RAS-mutated, *n* (%)	9 (64.3)
Order of resection, *n* (%)	
Bowel first	5 (35.7)
Liver first	3 (21.4)
Simultaneous resection	6 (42.9)

IQR, interquartile range; CLMs, colorectal liver metastases; CEA, carcinoembriogenic antigen; FU, fluoro-uracil; VEGF, vascular endothelial growth factor; EGFR, endothelial growth factor receptor

### Digestion and cell isolation

Macrophages (TAMs) were FACS sorted from the peritumoral areas of surgically resected CLM of 14 patients. Single-cell suspensions were obtained by manually mincing tissue into small fragments and incubating them for 1 h at 37 °C in Hanks' Balanced Salt Solution (HBSS; Euroclone) with 1 mg/ml Type IV Collagenase (Sigma-Aldrich), 2% fetal bovine serum (FBS; Sigma-Aldrich), 50 μg/ml DNase I (Sigma-Aldrich), and 10 mM Hepes (Lonza). The resulting cell suspension was filtered through a 100-μm cell strainer and erythrocytes were lysed with 1X BD Lysing Buffer (BD Biosciences). Cells were then incubated with a blocking solution containing 1% human serum in saline solution and stained with the following fluorophore-conjugated primary antibodies: anti-CD45 (BD Biosciences; clone HI30), anti-CD11b (BD Biosciences; clone ICRF44), anti-CD16 (BioLegend; clone 3G8), anti-CD14 (BD Biosciences; clone M5E2), anti-CD66b (BioLegend; clone G10F5), and anti-CD163 (BD Biosciences; clone GHI/61). Fixable Viability Stain 700 fluorescent dye (BD Biosciences) was used for dead cell exclusion. SYTO 16 Green Fluorescent Nucleic Acid Stain (ThermoFisher) was used to identify nucleated cells. Large and small TAMs (20,000 cells/type) were FACS sorted on a FACSAria III (BD Biosciences) following the gating strategy as previously published [4]. The freshly sorted L- and S-TAMs were promptly subjected to snap-freezing with liquid nitrogen to maintain the metabolite profiles of the samples. Subsequently, they were stored at − 80 °C until the metabolomic analysis was carried out. The tumoral tissue of the same patients was digested using the MACS tumor dissociation kit (Miltenyi Biotec), according to manufacturer protocol. Briefly, the MACS tumor dissociation kit enzyme mix (300 μl) was added to each sample. Next, samples were put into the gentleMACS Dissociator and digested using the tumor program. The cell suspension was then applied to a 100-μm cell strainer. Cells were counted and cultured in RPMI 1640 (Euroclone), supplemented with 20% of FBS (Euroclone), 1% non-essential amino acids (NEAA, Lonza), 1% glutamine (Lonza), and 1% penicillin/streptomycin (Sigma–Aldrich) in a 37 °C, 5% CO2 incubator.

### Isolation of human monocytes

Buffy coats were obtained from anonymized healthy donors (Humanitas Hospital) approved for *in-vitro* research. Monocytes were isolated by density gradient centrifugation using pluriMate centrifuge tubes (Pluriselect), after incubation with anti-human CD14 M-pluriBead (Pluriselect), according to the manufacturer's instructions. Isolated monocytes were washed, resuspended in phosphate-buffered saline (PBS) 1x^−/−^ and seeded in 6-well plates for 1 h in an incomplete RPMI-1640 medium without FBS, at a concentration of 2 × 10^6^ cells for each well to promote their adhesion.

### In vitro differentiation and stimulation of M1/M2 macrophages

The isolated monocytes were cultured for 5 days in RPMI 1640, supplemented with 10% FBS and 25 ng/ml of Recombinant Macrophage Colony-Stimulating Factor (M-CSF, PeproTech, Rocky Hill, USA) to generate M0 macrophages. After 5 days, macrophages were polarized in vitro toward M1 or M2 phenotypes. For M1-like polarization, macrophages were cultured in RPMI-1640 medium supplemented with 100 ng/ml lipopolysaccharides (LPS from *E. coli*; Sigma-Aldrich) and 50 ng/ml interferon-gamma (IFN-γ; PeproTech) and incubated for 18 h. Meanwhile, for M2-like polarization macrophages were cultured in a complete RPMI-1640 medium supplemented with 20 ng/ml interleukin-4 (IL-4; PeproTech), for 18 h.

The culture medium of M2 macrophages was supplemented with tumor necrosis factor-alpha (TNFα; 20 ng/ml; PeproTech) and Transforming Growth Factor-beta (TGFβ; 10 ng/ml; PeproTech) for 18 h as previously reported [18].

### Gene expression analysis

Gene expression analysis was performed on freshly sorted and *in-vitro* differentiated macrophages by *q*RT-PCR. Briefly, mRNA was extracted using a commercial kit (Total RNA Purification Kit, NORGEN) and quantified using NanoDrop™ 2000c spectrophotometer (Thermo Fisher Scientific). Subsequently, 500 ng of mRNA were reverse transcribed in cDNA using the High-Capacity RNA-to-cDNA™ Kit (Applied Biosystems™). The *q*RT-PCR was performed on selected genes for M1 and M2 macrophages, using SYBR green PCR master mix (Applied Biosystems™) and custom-designed primers (Sigma-Aldrich), listed in Table S1. The *q*RT-PCR was carried out using a 7900HT Fast Real-Time PCR System (Applied Biosystems™). The experiments were carried out in triplicate for each condition. The gene expression was normalized using glyceraldehyde-3-phosphate dehydrogenase (GAPDH) as housekeeping and determined using the 2^−ΔCT^ or 2^−ΔΔCT^ method.

### Metabolites extraction

Metabolites in L- and S-TAMs pellets (*n* = 20.000 cells/condition) were extracted by adding on the top of the cells pellets 25 µL cold MeOH (1:4), suspensions were incubated for 20 min at − 80 °C, and then centrifuged at 13,000 *g* × 15 min. The supernatant was stored at − 80 °C until the untargeted and targeted metabolomics analysis.

### Untargeted metabolomics

Flow Injection Analysis High-resolution mass spectrometry (FIA-HRMS) was used for untargeted metabolomics [19]. A portion of the metabolites extract (8 μL) was analyzed by -Orbitrap QExactive Mass Spectrometer (ThermoFisher Scientific) equipped with an electrospray source operated in negative and positive modes. Each run was carried out by injecting 8 μL of sample extract at a flow rate of 50 μL/min (Agilent 1200 Series) of mobile phase consisting of isopropanol/water (60:40, v/v) buffered with 5 mM ammonium at pH 9 for negative mode and methanol/water (60:40, v/v) with 0.1% formic acid at pH 3 for positive mode. Reference masses for internal calibration were used in continuous infusion during the analysis (m/z 210.1285 for positive and m/z 212.0750 for negative ionization). Mass spectra were recorded from m/z 50 to 1.000 with 60.000 resolutions. The source temperature was set to 240 °C with 25 L/min drying gas and a nebulizer pressure of 35 psig. MS/MS fragmentation pattern of the significant features was collected and used to confirm metabolite identity. All data processing and analysis were done with MATLAB R2016a (The Mathworks) using our in-house developed script [20].

### Targeted metabolomics of central metabolism

The targeted approach investigated the abundance of 83 metabolites (32 amino acids and derivatives, 19 nucleic acid-related compounds, 13 vitamins, 4 sugars, and 18 intermediates of glycolysis and tricarboxylic acid cycle (TCA cycle) by using liquid chromatography coupled with a triple quadrupole mass spectrometry system (LCMS-8060, Shimadzu). One (1) μL of metabolites extract was injected into a Discovery HS F5-3 (2.1 mm I.D. × 150 mm, 3 µm) column (Sigma-Aldric) using a 20-min gradient from 0 to 95% B (Acetonitrile), A (10 mM NH_4_HCO_2_ pH 3.5) at 350 µL/min. The LCMS-8060 mass spectrometer was equipped with an ESI source operating in both positive and negative ion and selected reaction monitoring (SRM) modes. The transitions identified during the optimization of the method are reported in Table S2. The MS settings were as follows: nebulizing gas flow rate: 3.0 L/min; drying gas flow rate: 15.0 L/min; DL Temperature: 250 °C; block heater temperature: 400 °C. Peak areas were automatically integrated using LabSolution Insight LC–MS (Shimadzu). Retention times and area ratio between quantifier and qualifier ions of all metabolites of interest were validated using pure standards.

### LSD1 activity assay

Lysine-specific demethylase 1 (LSD1) enzyme activity was evaluated using the Abcam KDM1/LSD1 Activity Quantification kit (ab113459, Abcam) according to the manufacturer’s protocol. Briefly, LSD1 protein was extracted from both the nuclei of S-L TAM pairs (8 subjects) and in-vitro M1 and M2 polarized monocytes. Nuclei were isolated using a Qproteome Nuclear Protein Kit (Qiagen) following the manufacturer’s protocol. Soluble protein extracts were then subjected to the KDM1/LSD1 activity kit.

### Immunohistochemistry

Formalin-fixed and paraffin-embedded specimens (2-μm thick tissue section) of CLM and peritumor tissue were deparaffinized and rehydrated in PBS. Antigen retrieval was performed by heat treatment using an ethylenediaminetetraacetic acid (EDTA) buffer (Dako; 0.25 mM, pH 8) in a water bath at 98 °C for 20 min. After washing with PBS, endogenous peroxidases were blocked via incubation with the Peroxidase Blocking Solution (Dako) for 15 min at room temperature (RT), and subsequently to block nonspecific binding, the slides were incubated with Background Sniper (Biocare Medical) for 20 min. After washing with PBS, the sections were then incubated with the primary antibody anti-human CD163 (Leica Biosystems, 10D6 clone, diluted 1:200) overnight at RT. The slides were washed twice with PBS and then incubated with the detection system EnVision + System HRP-labelled anti-mouse (Dako) for 1 h at RT. Following this, diaminobenzidine tetrahydrochloride (Dako) was used as a chromogen to visualize the positive cells. Nuclei were lightly counterstained with a Haematoxylin solution (Dako). The sections were dehydrated and mounted with a mounting medium (Eukitt).

### Statistical analysis

Continuous variables are presented as a range with a median, and discrete variables are presented as a number and percentage. The non-parametric Wilcoxon Mann–Whitney test or Kruskal–Wallis test was used when comparing two or more than two groups, respectively. The Spearman correlation analysis was used to assess the correlation between metabolites and L-, S-TAM morphologies. A *p* value * < 0.05, and ** < 0.01 were considered statistically significant for all tests. Computations were conducted using the software IBM-SPSS (v. 28, IBM) and GraphPad Prism (v. 9.2, Dotmatics).

## Results

### Morphological assessment confirmed small (S) and large (L)-TAMs in human CLM

The sorting strategy described by Donadon et al. was applied in this work to isolate large and small macrophages from the human peritumoral liver tissue of patients surgically resected for CLM [4]. The morphological assessment confirmed that S-, and L-TAMs were characterized by a different expression of CD163 (Fig. S1A), moreover, the distinct flow cytometry scatter plots of S-and L-TAMs characteristics resemble their morphologic aspect as previously reported for CLM patients (Fig. S1B), where L-TAM correlated to worse prognosis [4, 9].

### In vivo CLM-derived TAM populations are metabolically equivalent

**Fig. 1:**
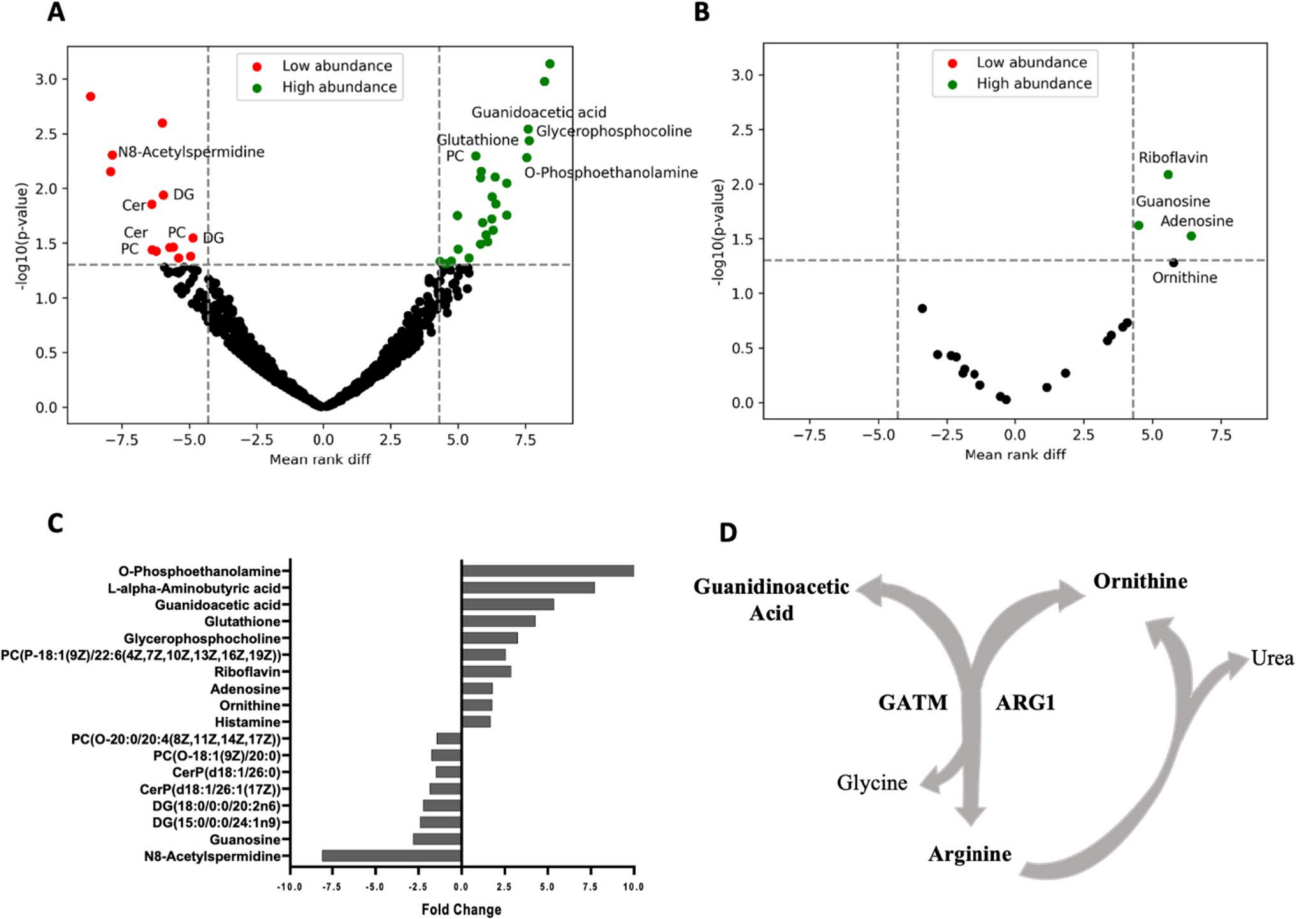
Untargeted and targeted mass spectrometry-based metabolomics highlight the similarity of metabolic landscape between small and large TAMs. **A** Volcano plot of the identified metabolic features (m/z) by untargeted metabolomics. The highlighted area identifies the statistically significant (Wilcoxon Mann–Whitney test, *p < 0.05) metabolites between L and S-TAM populations obtained from the 14 CLM patients. Dot colors indicated the trend of abundance between L- versus and S-TAMs. **B** Volcano plot of the detected metabolites by targeted metabolomics. The highlighted area identifies the statistically significant (Wilcoxon Mann–Whitney test, *p* values < 0.05) metabolites between L- and S-TAM populations obtained from the 14 CLM patients. Dot colors indicated the trend of abundance between L- versus S-TAMs. **C** Fold change of abundance of the statistically different metabolites between L- and S-TAMs determined using untargeted and targeted metabolomics data. **D** Schematic overview of the metabolic circuit linking arginine, ornithine, and guanidoacetic acid

To identify TAM features associated with the two distinct macrophage subpopulations, we conducted a wide metabolic profiling (untargeted and targeted mass spectrometry-based metabolomics analyses) on freshly isolated L- and S-TAM pairs derived from the 14 CLM patients. This analysis was aimed at elucidating the possible metabolic alterations underlying the in vivo TAM polarization and morphology in CLM. Due to difficulties in macrophage isolation from freshly resected peritumoral tissue, 20.000 cells were identified as the adequate number to perform a complete metabolic analysis. After cell sorting, the S- and L- TAMs were analyzed by untargeted profiling using the HMDB database. We identified 1380 and 270 m/z features in positive and negative ion mode respectively of whom 14 were statistically different (Wilcoxon Mann–Whitney test, *p* values < 0.05) in their abundance between the two macrophage groups (Fig. 1A**)**. Metabolites identity by MS/MS analysis was confirmed for 7 of them (Table S3): one amine (Histamine), three amino acids and derivatives (guanidoacetic acid, alpha-aminobutyric acid, and glutathione), one phosphate ester (O-Phosphoethanolamine), one carboximidic acid (N8-Acetylspermidine) and one glycerophosphocholine. Furthermore, the 7 statistically significant different m/z features, corresponding to lipid species, the identification could not have been established, given the existence of isomeric species with indistinguishable fragmentation spectra and the lack of reference standards. For these molecules, it was only possible to attribute the metabolic class to which they belong and not the exact chemical identity. The identified classes are two diacylglycerols (DG), two ceramide phosphates (CerP), and two phosphatidylcholines (PC) (Fig. 1A, Table S3). We further investigated, by using a targeted metabolomics approach, alteration in metabolites belonging to central cellular pathways such as glycolysis, the TCA cycle, the pentose phosphate pathway (PPP), and the urea cycle. The targeted approach quantified 48 out of the 83 measurable metabolites (Table S4) and indicated that L- and S-TAMs populations had a comparable central cellular metabolism with no substantial difference in the presence, an abundance of metabolites related to glycolysis, TCA cycle, urea cycle. We observed that only 4 metabolites, two purine nucleosides (Adenosine, Guanosine), a water-soluble vitamin (Riboflavin), and a non-proteinogenic amino acid (Ornithine) statistically discriminated (Wilcoxon Mann–Whitney test, *p* values < 0.05) between L- and S- TAMs populations (Fig. 1B, Table S4). No significant enrichment of specific biochemical pathways was found by using MetaboAnalyst (a web server for metabolomics data interpretation) on merged untargeted and targeted significantly different metabolites.

Comprehensively, metabolomics profiling indicates that L-TAMs were characterized by an increased abundance of the majority of deregulated metabolites except a few lipid species decreasing relative to the S-TAMs (Fig. 1C). To note, the arginine catabolism, used to generate ornithine and guanidoacetic acid, was the only metabolic circuit representative of the L-TAMs relative to the S- counterparts, thus supporting the role of such metabolic phenotype already ascribed to TAMs metabolism (Fig. 1D) [21].

According to these results, we hypothesized that the L- and S-TAM subpopulations have a similar central metabolic landscape with few discriminating metabolic points that only partially recapitulate the metabolic observation obtained in the in vitro induced TAM populations.

### Riboflavin-LSD1 relationship in triggering TAM morphologies

The correlation analysis between TAM morphologies and the statistically significant metabolites (from untargeted + targeted metabolomics) pointed out that riboflavin was the only metabolite with the strongest positive correlation with L-TAM morphology (Spearman *r*: 0.7732; *p* value: 0.0022), while the remaining metabolites showed moderate significant correlation (Spearman *r*: 0.5–0.6) values (Table 2). Histamine, O-phosphoethanolamine, glutathione, and L-alpha-aminobutyric acid were excluded from correlation analysis because their abundance obtained with the untargeted approach was not confirmed through targeted analysis. Consistently, Riboflavin was also found to be the metabolite with the strongest association with TAM morphologies (linear regression; standardized beta: 0.721; *t* = 3.752; *p* value = 0.002).

**Table 2:** Spearman’s correlation coefficient *r* and related *p* value between TAMs population morphology (L- and S-TAMs) and metabolite levels (untargeted + targeted)

Metabolites	Spearman *r*	*p* value	Summary
Riboflavin	0.7732	0.0022	**
Guanidoacetic acid	0.6495	0.0079	**
N8-Acetylspermidine	− 0.5958	0.0034	**
PC(P-18:1(9Z)/22:6(4Z,7Z,10Z,13Z,16Z,19Z))	0.5552	0.0111	*
DG(18:0/0:0/20:2n6)	− 0.5552	0.0111	*
CerP(d18:1/26:1(17Z))	− 0.5283	0.0055	**
Glycerophosphocholine	0.4883	0.0211	*
Ornithine	0.3128	0.1197	ns
Adenosine	0.3846	0.0524	ns
PC(O-20:0/20:4(8Z,11Z,14Z,17Z))	− 0.3203	0.1271	ns
CerP(d18:1/26:0)	− 0.3361	0.0804	ns
PC(O-18:1(9Z)/20:0)	− 0.3712	0.0566	ns
Guanosine	− 0.4124	0.5	ns
DG(15:0/0:0/24:1n9)	− 0.4174	0.0533	ns

**Fig. 2:**
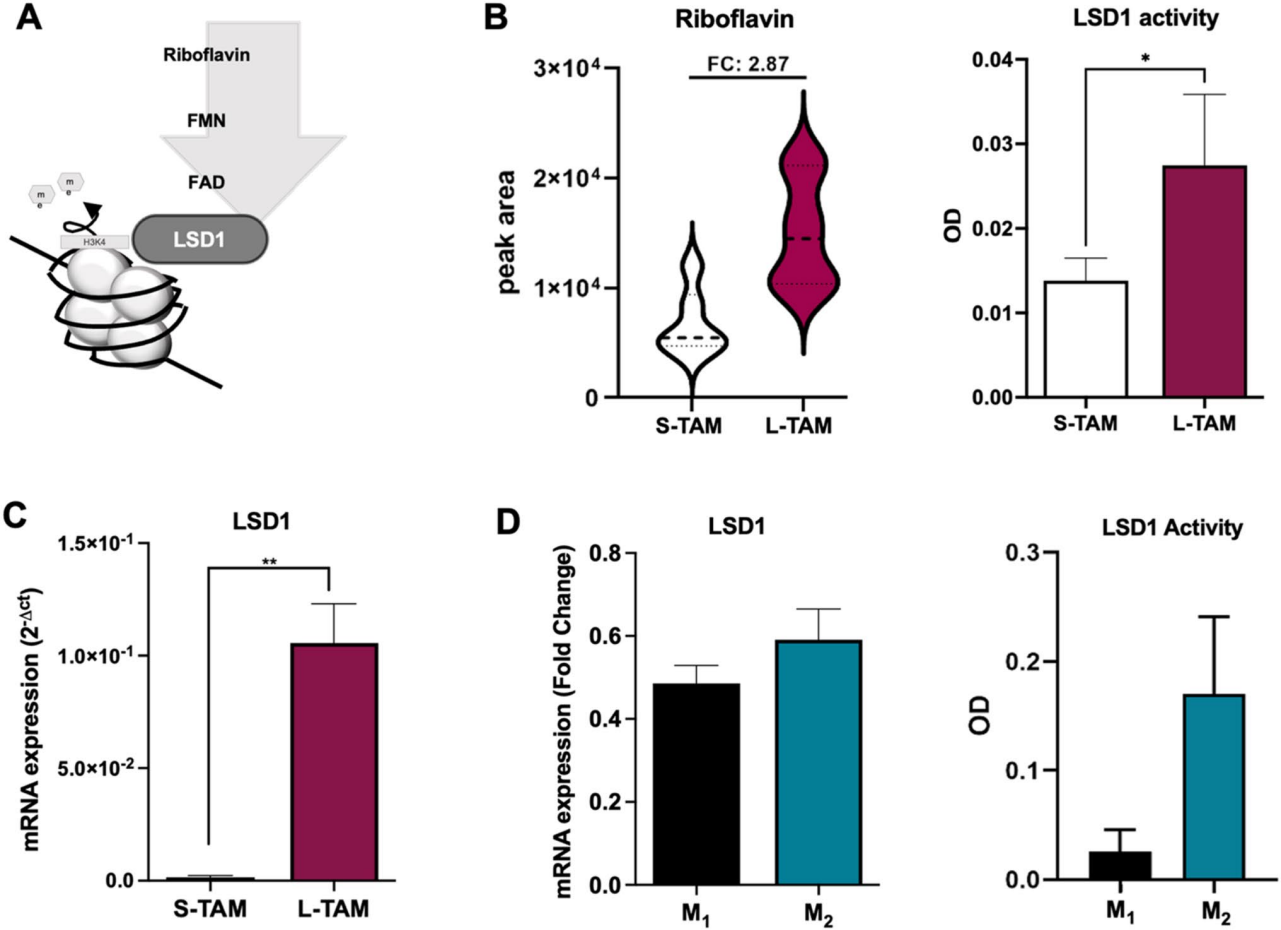
Role of riboflavin in driving TAMs morphology and polarization. **A** Schematic overview of the biochemical link between Riboflavin (vitamin B2) and LSD1 enzyme. **B** At left, riboflavin levels expressed as peak area in S- and L-TAM populations in the 14 TAM pairs. At right, LSD1 activity of S- and L-TAM pairs obtained from 8 CLM-patients expressed as OD. **C** LSD1 expression analyses in S- and L-TAMs (*n* = 4 CLM patients). Gene expressions were normalized using GAPDH as a housekeeping gene. **D** At left, LSD1 expression levels in M1 and M2 in-vitro polarized monocytes. At right, LSD1 activity of M1 and M2 polarized monocytes expressed as OD. Gene expressions were normalized using GAPDH as a housekeeping gene. Gene expression data are represented as mean ± SD of the average of three biological replicates. *p < 0.05, **p < 0.01 Wilcoxon Mann–Whitney test

Riboflavin, also known as Vitamin B2, has an important role in cell metabolism linked to the generation of flavin mononucleotide (FMN) and flavin adenine dinucleotide (FAD) cofactors. In literature, riboflavin has been described to be able to directly interact with the flavoprotein LSD1 (lysine-specific histone demethylase) by itself or FAD (Fig. 2A**)**, thereby influencing macrophages polarization toward the M2-like phenotype [22, 23]. In agreement with the increased riboflavin abundance, L-TAMs showed higher activity of LSD1 protein compared to S-TAMs (Fig. 2B). To corroborate this data, a *q*RT-PCR was performed on S-TAMs and L-TAMs sorted from the peritumoral samples (*n* = 4). The LSD1 expression was found to be significantly increased in the L-TAMs, compared to S-TAMs **(**Fig. 2C**).**

To understand the interplay between the Riboflavin-LSD1 axis and the different activation states of macrophages, the monocytes were differentiated in vitro into M1 and M2 macrophages. We observed a slightly higher expression and activity of LSD1 in the M2 macrophages, despite not being statistically significant, compared to the M1 macrophages (Fig. 2D). Overall, our data supported the involvement of the Riboflavin-LSD1 axis in driving the in vivo L-TAMs morphologies.

### Inflammatory cytokines are driving the riboflavin-LSD1 axis and L-TAMs morphology

To investigate how the interaction between tumor cells and macrophages may impact LSD1 expression and TAM morphologies, we analyzed the expression of inflammatory cytokines TNFα and TGFβ, which are released by tumor cells within the tumor microenvironment [24].

**Fig. 3:**
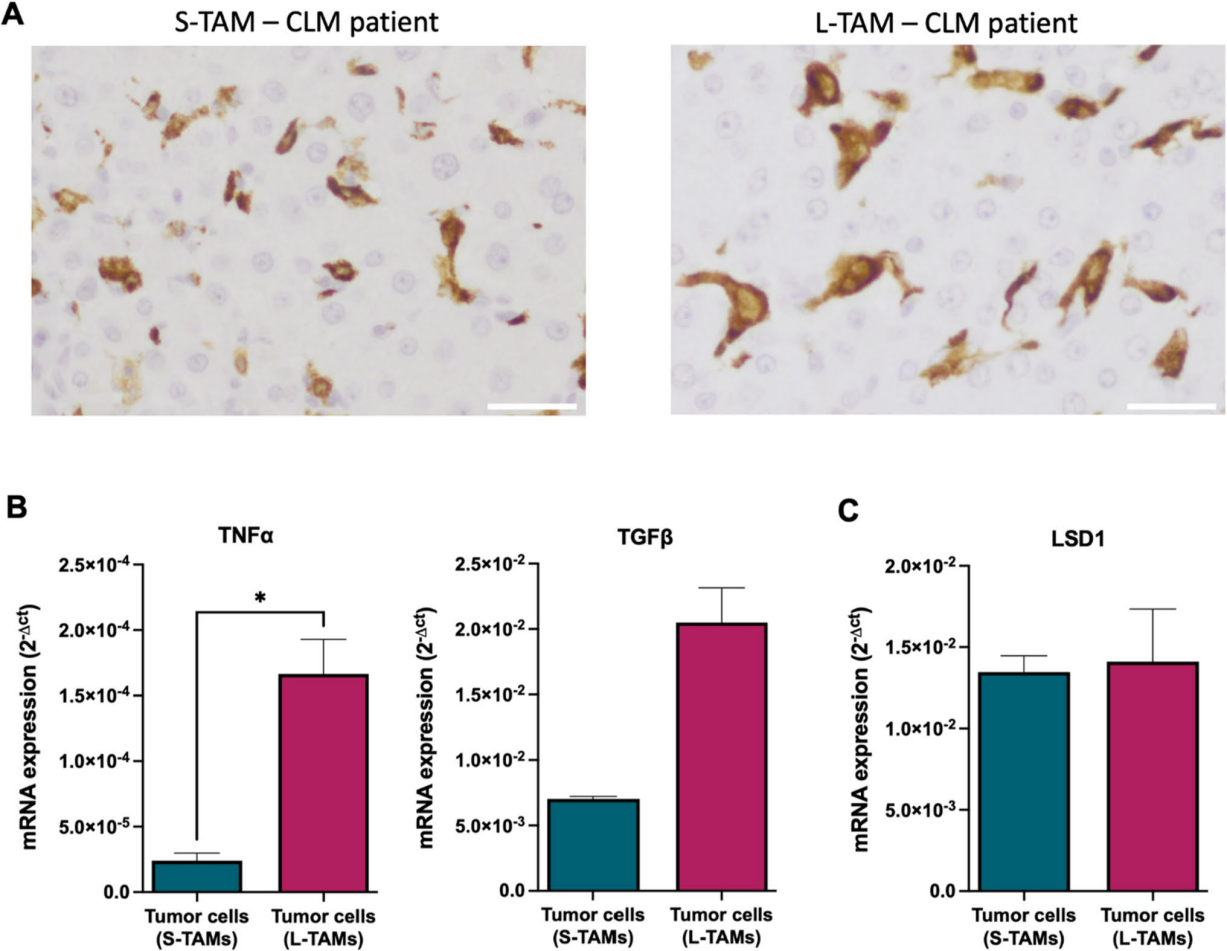
Gene analysis on patient-derived tumor cells highlighted high TNFα expression. **A** Representative immunohistochemistry images showing a CLM patient with higher S-TAMs (left) and higher LTAMs (right) in the peritumoral area. Scale bar: 30 µm. **B** TNFα and TGFβ expression in primary cells from patients with higher S-TAMs (*n* = 3) and with higher L-TAMs (*n* = 3). **C** LSD1 expression in primary cells from patients with higher S-TAMs (*n* = 3) and with higher L-TAMs (*n* = 3). Gene expressions were normalized using GAPDH as a housekeeping gene. Data are represented as mean ± SD of the average of three biological replicates (Mann–Whitney test; *p < 0.05)

According to the immunohistochemistry criteria previously reported [4, 9], we then selected 3 patients exhibiting high density of L-TAMs and 3 patients exhibiting low density of L-TAMs and high density of S-TAMs to isolate the corresponding primary tumor cells (Fig. 3A). Of note, the baseline demographic and tumor characteristics of these two groups of patients were statistically similar (Table S5), while we found an increase of TNFα and TGFβ in those patients with increased density of L-TAMs (Fig. 3B) with no differences in the LSD1 expression among these primary tumor cells (Fig. 3C).

**Fig. 4:**
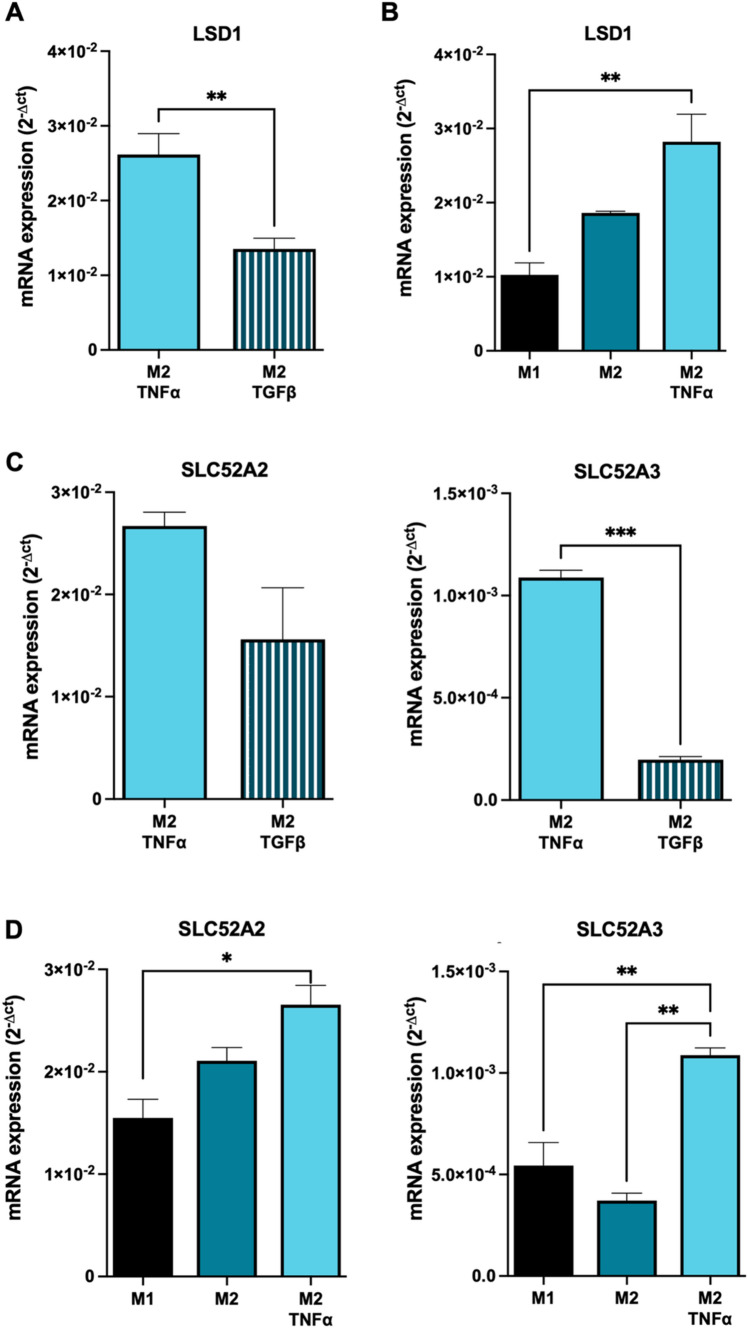
LSD1 and riboflavin receptors are increased in M2 macrophages under inflammatory stimuli. **A** LSD1 expression in monocytes differentiates into M2 macrophages exposed to TNFα (20 ng/ml) for 18 h. **B** LSD1 expression in monocytes differentiates into M2 macrophages exposed to TGFβ (10 ng/ml) for 18 h. **C** Riboflavin receptors (SLC52A2 and SLC52A3) expressions in M2 macrophages exposed to TNFα (20 ng/ml) or TGFβ (10 ng/ml) for 18 h. **D** Riboflavin receptors (SLC52A2 and SLC52A3) expressions in M1, M2, and M2 exposed to TNFα (20 ng/ml) or TGFβ (10 ng/ml) for 18 h. Gene expressions were normalized using GAPDH as a housekeeping gene. Data are represented as mean ± SD of the average of three biological replicates. **p < 0.01; ***p < 0.001 Mann–Whitney test when comparing two groups. *p < 0.05; **p < 0.01 One-way ANOVA when comparing three groups

To elucidate whether the TNFα and TGFβ released from primary tumor cells could be involved in the determination of L-TAMs morphology, the culture medium of the in vitro-polarized M2 macrophages was supplemented with TNFα and TGFβ for 18 h. As shown in Fig. 4A, M2 macrophages cultured in the presence of TNFα displayed a significantly higher LSD1 expression, compared to those exposed to TGFβ, suggesting the crucial role of TNFα in the upregulation of LSD1 in L-TAMs. Furthermore, when exposed to TNFα, M2-induced macrophages exhibited a significant upregulation of LSD1 expression compared to M1 macrophages, mirroring the observed differences in the LSD1 expression between S- and L-TAMs (Fig. 4B).

To gain insight into the biochemical link between riboflavin and LSD1 in the presence of inflammatory stimuli, we evaluated the expression of the two riboflavin membrane transporters, namely Solute Carrier Family 52 Member 2 (SLC52A2) and SLC52A3.

In agreement with the LSD1 upregulation, the M2 macrophages exposed to TNFα showed increased expression of SLC52A3 and SLC2A2 (although the latter did not reach statistical significance) compared to M2 macrophages cultured with TGFβ (Fig. 4C). Additionally, similar to the findings observed for LSD1 expression, TNFα-treated M2 macrophages exhibited a significantly increased expression of SLC52A3, compared to M1 macrophages (Fig. 4D).

These results suggest a collaborative role of Riboflavin and LSD1 in shaping the morphology of the L-TAMs within an inflammatory microenvironment influenced by tumor cells. Moreover, it is suggested that SLC52A3, one of the riboflavin membrane transporters, plays a direct role in the intracellular uptake of riboflavin, thereby contributing to this mechanism.

## Discussion

TAMs are crucial elements of the tumor microenvironment, and their clinical relevance in predicting patient outcomes has been reported in large cohorts of different cancer patients [25]. TAMs are characterized by great plasticity and adaptability making difficult their characterization. Few articles dissected the metabolic characteristics of the in vivo human TAM populations, and up to now, it is only possible to state that in vivo patient-derived TAM populations had an augmented metabolic activity that did not wholly recapitulate the M1, M2 macrophages metabolic peculiarity [26].

Here, we described for the first time in a limited but consistent population of (14 L-TAMs and 14 S-TAMs) the snapshot of the metabolic state of human TAMs in CLMs, by dividing them according to their dimension and appearance, namely L- and S-TAM subpopulation, being such diversity of prognostic significance [4, 9].

We assessed that L- and S-TAMs are characterized by high metabolic variability. Our findings align with existing literature data that indicated heterogeneous metabolic profiles of TAMs, primarily related to central cellular metabolism, without distinct metabolic characteristics [8, 13–15, 27].

In this study, we uncovered the involvement of the Riboflavin-LSD1 biochemical axis in triggering in vivo TAM morphologies. Furthermore, we supported and validated the crucial role of riboflavin in activating LSD1 protein by prompting the protein expression and activity, both in the in vivo sorted L-TAMs and in vitro differentiated M2 macrophages [22, 23].

Given the widely acknowledged role of the inflammatory microenvironment as a hallmark of cancer progression and the continuous interaction between TAMs and tumor cells, akin to the interplay between macrophages and their surrounding parenchyma during homeostasis [28], we have successfully demonstrated the connection between inflammatory stimuli and the modulation of TAMs morphologies. TNFα, an inflammatory cytokine known to be involved in the modulation of numerous signaling pathways [29] and exhibiting dysregulated expression in several neoplastic diseases [30], displayed a remarkable seven-fold increase in primary cancer cells derived from patients with a higher number of L-TAMs compared to S-TAMs.

Consistent with the elevated intracellular levels of riboflavin in L-TAMs, the presence of TNFα in the culture medium of the in-vitro differentiated M2 macrophages promoted the upregulation of riboflavin transporters, likely enabling the intracellular uptake of riboflavin into L-TAMs, thereby triggering the activation cascade ruled by the LSD1 protein.

The specific mechanism by which TNFα triggers the enhanced expression of riboflavin transporters has not yet been fully understood and requires further elucidation. Nevertheless, the detrimental role of riboflavin and its associated transporters, namely SLC52A2 and SLC52A3, in cancer has been extensively characterized. Indeed, these transporters have been described to play a critical role in several tumor aspects such as immune evasion, and macrophage M2 polarization, and their expression has been associated with advanced tumor stages [15, 23, 31].

We acknowledge some limitations in our study mainly derived from our focus on avoiding experimental manipulation of the sorted TAM culture to ensure a precise assessment of their in vivo metabolic assets. Considering the clinical and experimental efforts needed to obtain L- and S-TAMs population from CLM specimens (usually there are less than 20.000 TAM cells per specimen), we limited the number of clinical samples to 14 human CLMs that should be considered adequate for a preliminary investigative study. This relatively small sample size has hampered the possibility of performing further molecular and cellular experimental purposes. Nevertheless, to support our results we did an external validation study involving in vivo polarized macrophages that supported the in vivo obtained results, such as the increased protein activity in the M2 phenotypes as observed in the human L-TAMs. Moreover, our findings proved that TAMs exhibiting elevated riboflavin content and increased LSD1 expression correspond to the previously described L-TAM population in CLMs [4], primarily localized in the peritumoral region [9].

In conclusion, we supported the paramount importance of the LSD1 protein in orchestrating the L-TAM morphologies, in agreement with its previously described role in the M1/M2 phenotype transition, which raises the potential use of LSD1 inhibitors to induce macrophage reprogramming [22]. In such a scenario, this approach holds promise for potentially priming the S-TAMs phenotype in CLM and other cancer types. Since TAMs play a significant role as a cell-extrinsic factor contributing to the tumor cell resistance to chemotherapy or radiotherapy, our findings suggest that riboflavin and LSD1 could represent potential biomarkers for these cells. Furthermore, the selective inhibition of LSD1 function in L-TAMs could be a promising strategy to enhance cancer treatments and TAMs reprogramming from an anti-inflammatory to an anti-tumor phenotype, thereby paving the way for novel therapeutic interventions.

### Supplementary Information

Below is the link to the electronic supplementary material.

Supplementary file1 (DOCX 12 kb)

Supplementary file2 (XLSX 14 kb)

Supplementary file3 (XLSX 11 kb)

Supplementary file4 (XLSX 16 kb)

Supplementary file5 (DOCX 14 kb)

Supplementary file6 (DOCX 1351 kb)
