# Supplementary material for: Riboflavin-LSD1 axis participates in the in vivo tumor-associated macrophage morphology in human colorectal liver metastases
Source: Cancer Immunol Immunother. 2024 Mar 2;73(4):63. doi: 10.1007/s00262-024-03645-1 (PMC10908638; doi:10.1007/s00262-024-03645-1)
Supplement: Supplementary file 1 — Supplementary file1 (DOCX 12 kb) [file 262_2024_3645_MOESM1_ESM.docx]

**Table S1:** Sequence of primers used for RT-PCR studies.

| **Gene** | **Forward Primer (5’-3’)** | **Reverse primer (5’-3’)** |
| --- | --- | --- |
| **GAPDH** | CAGTCAGCCGCATCTTCTTT | TTGACTCCGACCTTCACCTT |
| **LSD1** | AGATGGATGAAAGCTTGGCC | AGGAAGTCGGCTCTGGAAAG |
| **TNFα** | TGTTCCTCAGCCTCTTCTCC | GAGGGCTGATTAGAGAGAGGT |
| **TGFβ** | AAGACTTTTCCCCAGACCT | CTCAGTATCCCACGGAAATA |
| **SLC52A2** | CGACTTCCTTGAGCGTTTTC | GCAACAGCAGCAGAAGACC |
| **SLC52A3** | CTGCTCATGTTCCCTCTGGT | CTCTCTGTTCCTGCCCCTTG |
