# Supplementary material for: Riboflavin-LSD1 axis participates in the in vivo tumor-associated macrophage morphology in human colorectal liver metastases
Source: Cancer Immunol Immunother. 2024 Mar 2;73(4):63. doi: 10.1007/s00262-024-03645-1 (PMC10908638; doi:10.1007/s00262-024-03645-1)
Supplement: Supplementary file 5 — Supplementary file5 (DOCX 14 kb) [file 262_2024_3645_MOESM5_ESM.docx]

**Table S5**: Demographic and clinical characteristics at baseline of the CLM patients with high L-TAMs and high S-TAMs

| **VARIABLES** | **L-TAM**  **N=3** | **S-TAM**  **N=3** |
| --- | --- | --- |
| **Sex, male**, n (%) | 1 (33.3) | 3 (100.0) |
| **Age, year**, median (IQR) | 71.8 (59.1 – NA) | 70.1 (43.7 – NA) |
| **Size of CLMs**, median (IQR) | 3.5 (2.0 – NA) | 6.5 (3.5 – NA) |
| **Number of CLMs**, median (IQR) | 15.0 (7.0 – NA) | 3.0 (1.0 – NA) |
| **Bilobar disease**, n (%) | 3 (100.0) | 1 (33.3) |
| **Preoperative CEA (ng/ml)**, median (IQR) | 230.8 (188.5 – NA) | 29.5 (9.4 – NA) |
| **Preoperative CA19.9 (IU/ml)**, median (IQR) | 138.3 (7.1 - NA) | 17.5 (13.5 – NA)) |
| **Grading of the primary tumor,** n (%)  G2-G3 | 2 (66.7) | 1 (33.3) |
| **Staging of the primary tumor**, n (%)  T3-4  N+ | 1 (33.3)  0 (0.0) | 1 (33.3)  2 (66.7) |
| **Synchronous presentation**, n (%) | 3 (100.0) | 2 (66.7) |
| **Site of the primary tumor**, n (%)  Colon  Rectum | 3 (100.0)  0 (100.0) | 2 (66.7)  1 (33.3) |
|  |  |  |
|  |  |  |
| **RAS-mutated**, n (%) | 3 (100.0) | 1 (33.3) |
| **Order of resection**, n (%)  Bowel first  Liver first  Simultaneous resection | 0 (0.0)  2 (66.7)  1 (33.3) | 2 (66.7)  0 (0.0)  1 (33.3) |
| **Disease-free survival,** mean (95%CI) | 3.5 (0.0-7.5) | 14.3 (0.3-28.5) |

IQR= interquartile range, CLMs= colorectal liver metastases, CEA=carcinoembriogenic antigen, FU= fluoro-uracil, VEGF= vascular endothelial growth factor, EGFR= endothelial growth factor receptor
