# Supplementary material for: Riboflavin-LSD1 axis participates in the in vivo tumor-associated macrophage morphology in human colorectal liver metastases
Source: Cancer Immunol Immunother. 2024 Mar 2;73(4):63. doi: 10.1007/s00262-024-03645-1 (PMC10908638; doi:10.1007/s00262-024-03645-1)

**Riboflavin-LSD1 axis participates in the in vivo tumor-associated macrophage morphology in human colorectal liver metastases**

Cristiana Soldani1*, Giulia De Simone2,3*, Michela Anna Polidoro1, Aurelia Morabito2,4, Barbara Franceschini1, Federico Simone Colombo5, Achille Anselmo5, Flavio Milana6,8, Ana Lleo6,7, Guido Torzilli6,8, Roberta Pastorelli2*, Matteo Donadon1,8,9,10§, Laura Brunelli2§

**Supplementary Table legend**

**Supplementary Table 1:** Sequence of primers used for RT-PCR studies.

**Supplementary Table 2:** List of the Targeted metabolomics analysis

**Supplementary Table 3**: Statistically significant different metabolites in untargeted analysis

**Supplementary Table 4:** Targeted metabolomics analysis results

**Supplementary Figure**

**Figure 1.** An experimental approach to sort and differentiate S- and L-TAM populations in CLM patients. A) Small macrophages (S-TAMs) were sorted as alive CD45+/CD11b+/CD66b−/CD14dim/CD163dim/FSC-A low and large macrophages (L-TAMs) as CD45+/CD11b+/CD66b−/CD14dim/CD163hi/FSC-A high cells. B) Representative immunohistochemistry image of the CD163+ cells in the peritumoral area of CLM patients.


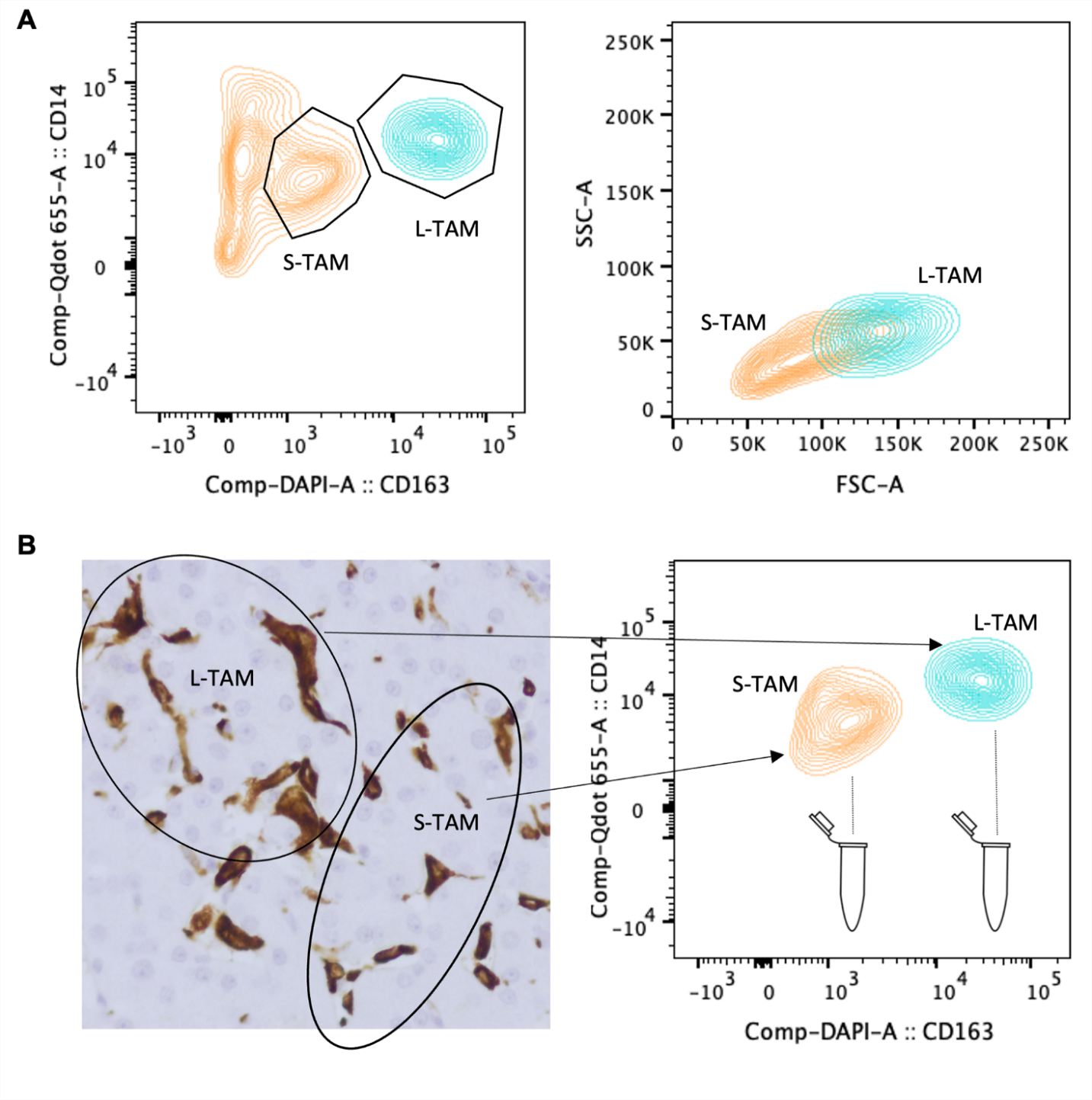

Supplement: Supplementary file 6 — Supplementary file6 (DOCX 1351 kb) [file 262_2024_3645_MOESM6_ESM.docx]
